# Fibrotic Lung Disease Alters Neutrophil Trafficking and Promotes Neutrophil Elastase and Extracellular Trap Release

**DOI:** 10.4049/immunohorizons.2200083

**Published:** 2022-12-01

**Authors:** Helen I. Warheit-Niemi, Gabrielle P. Huizinga, Summer J. Edwards, Yizhou Wang, Susan K. Murray, David N. O’Dwyer, Bethany B. Moore

**Affiliations:** * Department of Microbiology and Immunology, University of Michigan, Ann Arbor, MI; † Immunology Graduate Program, University of Michigan, Ann Arbor, MI; ‡ Department of Biostatistics, University of Michigan, Ann Arbor, MI; § Department of Internal Medicine, Division of Pulmonary and Critical Care Medicine, University of Michigan Medical School, Ann Arbor, MI

## Abstract

Idiopathic pulmonary fibrosis (IPF) is a progressive, irreversible disease characterized by collagen deposition within the interstitium of the lung. This impairs gas exchange and results in eventual respiratory failure. Clinical studies show a correlation between elevated neutrophil numbers and IPF disease progression; however, the mechanistic roles neutrophils play in this disease are not well described. In the present study, we describe alterations to the trafficking and function of neutrophils after the development of fibrosis. We observed increased numbers of total and aged neutrophils in peripheral tissues of fibrotic mice. This appeared to be driven by an upregulation of neutrophil chemokine *Cxcl2* by lung cells. In addition, neutrophil recruitment back to the bone marrow for clearance appeared to be impaired, because we saw decreased aged neutrophils in the bone marrow of fibrotic mice. Neutrophils in fibrosis were activated, because ex vivo assays showed increased elastase and extracellular trap release by neutrophils from fibrotic mice. This likely mediated disease exacerbation, because mice exhibiting a progressive disease phenotype with greater weight loss and mortality had more activated neutrophils and increased levels of extracellular DNA present in their lungs than did mice with a nonprogressive disease phenotype. These findings further our understanding of the dynamics of neutrophil populations and their trafficking in progressive fibrotic lung disease and may help inform treatments targeting neutrophil function for patients with IPF experiencing disease exacerbation in the future.

## INTRODUCTION

Idiopathic pulmonary fibrosis (IPF) is a chronic and irreversible disease affecting the interstitium of the lung. A characteristic feature of IPF is progressive collagen deposition by fibroblasts within the lung interstitium, resulting in impaired gas exchange and eventual respiratory failure. IPF is most common in older individuals, with a reported prevalence of 494.5 cases per 100,000 persons aged 65 and older (1). With the world’s older population steadily increasing, IPF is and will remain a concerning public health burden.

Although the cause of IPF remains unknown, disease is thought to result from an abnormal wound-healing response in the lung. Recurring microinjuries to alveolar epithelial cells or ongoing cellular stress are hypothesized to drive a cycle of inflammation characterized by release of damage-associated molecular patterns, production of cytokines, and recruitment of immune cells, resulting in an aberrant tissue repair program within the lung.

Many studies have focused on unraveling the role of specific cell types in IPF pathogenesis. Of particular interest is the function of immune cells in disease development; however, the role of neutrophils in fibrosis has remained particularly enigmatic. Clinical studies show that patients with IPF have elevated neutrophil numbers and neutrophil-to-lymphocyte ratios (NLRs) in blood and bronchoalveolar lavage fluid (BALF) compared with healthy control individuals and that increased neutrophil levels are correlated with poor disease outcomes (2-6).

However, the precise role of neutrophils in fibrotic disease progression is unknown. One hypothesis suggests that neutrophils are required for the initiating injury to the alveolar epithelial cells, driving the aberrant wound-healing response, and many studies have shown that inhibiting neutrophil migration to the lung after fibrotic lung injury inhibits the development of lung fibrosis (7-9). However, other studies have found that simply depleting neutrophils early (before or immediately after lung injury) does not abrogate eventual fibrosis development, suggesting a role for neutrophils later in the disease course (8, 10-12). To this end, multiple studies have shown that neutrophil products such as neutrophil elastase (NE) and neutrophil extracellular traps (NETs) contribute to the differentiation of myofibroblasts and activation of TGF-β, indicating that neutrophil activity may be involved in fibrogenesis and collagen deposition itself (13-17). Mice deficient in NE and mice treated with an NE inhibitor had reduced collagen deposition after fibrotic lung injury as well as decreased myofibroblasts present in the lungs and reduced levels of active TGF-β (15-17).

In the present study, we sought to understand neutrophil homeostasis at baseline and during fibrogenesis and hypothesized that fibrosis-induced alterations to neutrophil trafficking and function may perpetuate fibrotic disease. In this study, we show that, like clinical data from patients with IPF, bleomycin-induced lung fibrosis results in increased neutrophil numbers and enhanced levels of NE and extracellular DNA (exDNA) in BALF. Strikingly, we demonstrate that fibrosis alters the intrinsic function of neutrophils, resulting in increased release of NE and NETs ex vivo. Increased NE release is likely mediated in part by soluble factors present in the fibrotic lung microenvironment, such as CXCL2 and TGF-β, because treatment of naive neutrophils with BALF from fibrotic mice induced significantly more NE release than BALF from nonfibrotic mice. Last, we show that increased neutrophils are associated with worse outcomes in fibrotic mice. Our study strengthens the correlation between neutrophils and disease progression in IPF and provides insight into neutrophil functional alterations that may mediate exacerbation of fibrosis.

## MATERIALS AND METHODS

### Mice

Mouse studies were performed in accordance with the University of Michigan Institutional Animal Care and Use Committee–approved protocol. Six- to 8-wk-old male C57BL/6J mice were purchased from The Jackson Laboratory and housed in specific pathogen-free conditions. Mice were acclimated for 1 wk and then treated with bleomycin at 7–9 wk of age. For mortality and weight loss studies, criteria for euthanasia were defined as hunched and scruffy body condition, drop in body temperature to touch, limited mobility within cage, and greater than 25% starting body weight loss.

### Bleomycin

Bleomycin was obtained from the University of Michigan Hospital and was diluted in sterile PBS. Mice were treated with 50-μl volumes of bleomycin oropharyngeally at a concentration of 0.016 U per 20–25-g mouse. For most experiments, mice were euthanized 21 d after bleomycin treatment.

### In vivo anti-Ly6G treatment

Mice were injected i.p. with 500 μg anti-Ly6G (clone 1A8; Bio X Cell, Lebanon, NH) on day 12 after bleomycin and every 48 h after for a total of five doses of Ab treatment.

### Bronchoalveolar lavage

After euthanasia of each mouse, a catheter was inserted into the trachea, and the lungs were flushed with 2 × 1 ml of plain RPMI. BALF was centrifuged at 400 × *g* for 5 min to pellet cells for flow cytometry, and supernatant was collected and stored at −80°C.

### Blood collection, plasma isolation, and complete blood count (CBC)

Approximately 500 μl of blood was collected from each mouse via cardiac puncture and added to a tube containing 15 μl of 0.5 M EDTA for a final concentration of 16 μM EDTA. Warmed ammonium-chloride-potassium (ACK) lysis buffer (10 ml) was added to blood, incubated for 5 min, then neutralized with 20 ml of warmed PBS. Cells were pelleted at 400 × *g* for 10 min, and supernatant was aspirated. Cell pellet was resuspended in 5 ml ACK lysis buffer, incubated for 5 min, neutralized with 20 ml PBS, and pelleted as described. Isolated cells were then counted and stained for flow cytometry.

To isolate plasma, whole blood in 16 μM EDTA was centrifuged for 10 min at 1500 × *g*. The plasma layer was carefully removed from the RBC layer and stored at −80°C.

For CBC, 200 μl of whole blood was collected and added to EDTA Microtainer tubes (BD Biosciences, San Jose, CA). Samples were submitted to the University of Michigan In Vivo Animal Core for analysis.

### Bone marrow isolation

Femurs and tibias were collected, and the ends were removed from the bones. Whole bone marrow was flushed from bones with complete DMEM (DMEM + 10% FBS + 1% l-glutamine + 1% penicillin/streptomycin + 0.1% amphotericin) as previously described (18), after which cells were washed and RBCs were lysed with ACK lysis buffer.

### Total lung cell and macrophage preparation

Total lung cells were isolated via collagenase digest as previously described (19). Briefly, individual lung lobes were removed and finely minced with scissors, then incubated in DMEM containing 10% FCS, 1 mg/ml collagenase (MilliporeSigma, St. Louis, MO), and 30 μg/ml DNase for 35 min at 37°C. Cells were separated from lung tissue via mechanical dispersion, RBCs were lysed, and cells were centrifuged through a 20% Percoll gradient (Sigma-Aldrich/MilliporeSigma). Total lung macrophages were isolated from total lung cells via adherence purification for 1 h.

### Lung fibroblast isolation and fibroblast assays

To isolate primary lung fibroblasts, lungs were removed under aseptic conditions and minced with sterile scissors, then transferred to T175 cell culture flasks in complete DMEM. Cells were incubated for 2 wk, and media were changed every 3–4 d.

For proliferation assays, the MTT cell growth kit (Millipore-Sigma) was used. Fibroblasts were plated at 25,000 cells per well in 96-well plates in complete RPMI or conditioned media (CM) (supernatant from 4-h neutrophil cultures + 10% FBS + 1% l-glutamine + 1% penicillin/streptomycin + 0.1% amphotericin). Cells were incubated for 48 h. After 24 h, MTT reagent was added, and after 48 h, absorbance was read at 570 nm.

For gene expression, cells were plated at 250,000 cells per well in 12-well plates in CM or complete RPMI containing 4 ng/ml TGF-β (R&D Systems, Minneapolis, MN) for 24 h, after which media were removed, and cells were lysed in TRIzol for RNA isolation.

### Cell marker staining for flow cytometry

If Zombie Violet was used (Invitrogen/Thermo Fisher Scientific, Waltham, MA) for live/dead staining, cells were incubated with Zombie diluted 1:500 in PBS for 20 min at room temperature. If Zombie Violet was not used, this step was skipped. Cells were incubated with Fc block on ice for 15 min, then stained with combinations of the following Abs: Ly6G-FITC (catalog no. 561105), CD45-BV510 (catalog no. 563891) (BD Biosciences); CD11b-BV650 (catalog no. 101259) Ly6G-allophycocyanin (catalog no. 127613), CD62L-BV421 (catalog no.104435), CXCR4-PE/Dazzle (catalog no. 146513), Apotracker Green (catalog no. 427401) (BioLegend, San Diego, CA). Cells were incubated with Abs in the dark for 30 min on ice, after which they were washed two times with FA buffer (Difco/BD Biosciences). Cells were then fixed in 4% paraformaldehyde for 20 min on ice and washed again.

### NE, myeloperoxidase (MPO), exDNA, and citrullinated histone H3 (CitH3) in BALF and plasma

For elastase quantification, 5 μl of either cell-free BALF or plasma was added to a black-sided 96-well plate and combined with 95 μl Cell-Based Assay Buffer (Cayman Chemical, Ann Arbor, MI) and 10 μl of elastase-specific substrate (Z-Ala-Ala-Ala-Ala)2Rh110 (Cayman Chemical) for 1.5 h at 37°C. Upon cleavage by NE, (Z-Ala-Ala-Ala-Ala)2Rh110 yields fluorescence detectable at excitation/emission 485 nm/525 nm.

For MPO quantification in BALF, equal parts cell-free BALF and tetramethylbenzidine substrate solution (Cayman Chemical) were combined in a 96-well plate and incubated for 30 min. Absorbance of samples was then read at 650 nm.

For exDNA quantification, 5 μl of either cell-free BALF or plasma was diluted into 95 μl PBS + 0.1% BSA in a black-sided 96 well plate. Sytox Green (1 μM; Invitrogen/Thermo Fisher Scientific) in PBS + 0.1% BSA was added to samples, and the plate was immediately read at excitation/emission 485 nm/525 nm.

For CitH3 quantification, the CitH3 ELISA kit (Cayman Chemical) was used according to the manufacturer’s instructions.

### Bone marrow neutrophil isolation and ex vivo assays

Whole bone marrow was collected as described above, and cells were centrifuged through a Histopaque 1119/1077 gradient (Sigma-Aldrich/MilliporeSigma) at 900 × *g* (without brake) for 30 min to isolate neutrophils.

Neutrophils were plated at 100,000 cells per well and treated with the following: plain RPMI, BALF collected in plain RPMI diluted 1:1 with plain RPMI, plain RPMI + 10 ng/ml TGF-β (R&D Systems), plain RPMI + 100 ng/ml CXCL2 (R&D Systems), or plain RPMI + 10 ng/ml IL-17 (R&D Systems).

Ex vivo NE release was measured using the elastase-specific substrate (Z-Ala-Ala-Ala-Ala)2Rh110 (Cayman Chemical). Neutrophils were plated in 96-well plates in plain RPMI or 1:1 BALF and incubated for 4 h. Time points were taken after 1 h and 4 h, and supernatant was collected and incubated with elastase substrate for 1.5 h at 37°C. Fluorescence was then read at excitation/emission 485 nm/525 nm.

To measure ex vivo NETosis, neutrophils were seeded into black-sided 96-well plates and incubated in RPMI containing 0.2 μM Sytox Green (Invitrogen/Thermo Fisher Scientific), 5 μM ionomycin + 0.2 μM Sytox Green, or 1:1 BALF + 0.2 μM Sytox Green for 5 h. After incubation, an equal volume of 4% paraformaldehyde was added to samples and fixed overnight in the dark at 4°C. The following day, plates were read at excitation/emission 485 nm/525 nm.

### Hydroxyproline assay

After euthanasia, lungs were perfused with 3 ml sterile PBS. Lungs were either used immediately for assays or snap-frozen in liquid nitrogen and stored at −80°C until use. Lung tissue was homogenized in 1 ml sterile PBS with cOmplete protease inhibitor (Roche/MilliporeSigma). Hydroxyproline assays were performed as previously described (19).

### Bicinchoninic acid (BCA) assay, albumin ELISA, and E-selectin ELISA

The BCA assay was performed using the Pierce BCA Protein Assay Kit (Thermo Fisher Scientific) according to the manufacturer’s instructions. BALF was diluted 1:20–1:50 for BCA assays.

Albumin was quantified using a Mouse Albumin ELISA Kit (Bethyl/Fortis Life Sciences, Waltham, MA) according to the manufacturer’s instructions. BALF was diluted 1:10,000 for albumin ELISAs.

E-selectin was measured using the Mouse E-selectin/CD62E DuoSet ELISA (R&D Systems) according to the manufacturer’s instructions. BALF was diluted 1:2.5–1:4 for E-selectin ELISAs.

### RNA isolation and RT-qPCR

RNA was isolated using TRIzol reagent (Invitrogen/Thermo Fisher Scientific) according to the manufacturer’s instructions. One-step RT-qPCR was performed using the TaqMan RNA-to-Ct 1-Step Kit (Applied Biosystems/Thermo Fisher Scientific). Custom primers and probes (Table II) were purchased from Sigma-Aldrich.

### IPF cohort study

The survival of patients with IPF attending the interstitial lung disease (ILD) clinic at the University of Michigan was retrospectively analyzed. All patients discussed at the ILD multidisciplinary meeting were identified, and CBC and differential results within 6 mo of case presentation were obtained by medical chart review. The study was approved by the institutional review board at the University of Michigan. Kaplan-Meier estimates were used to study associations of time to death and time to death or first respiratory hospitalization. Hazard ratios (HRs) were determined by multivariate analysis using a Cox proportional hazards model. Statistical significance was determined after adjusting for age, sex, smoking status, and baseline forced vital capacity (FVC).

### Statistics

Statistical analyses were performed using GraphPad Prism software. Group sizes were determined on the basis of small pilot studies (*n* ≈ 3) followed by power analyses. For comparisons between two groups, unpaired Student *t* (parametric) or Mann-Whitney *U* (nonparametric) tests were used. For comparisons between three or more groups, one-way ANOVA (parametric) or Kruskal-Wallis (nonparametric) tests were used. Multiple comparisons posttests are specified in the figure legends. A *p* value < 0.05 was considered significant. Experiments were performed at least twice.

## RESULTS

### Neutrophil distribution across bone marrow, blood, and lung differs in fibrosis

**FIGURE 1.**
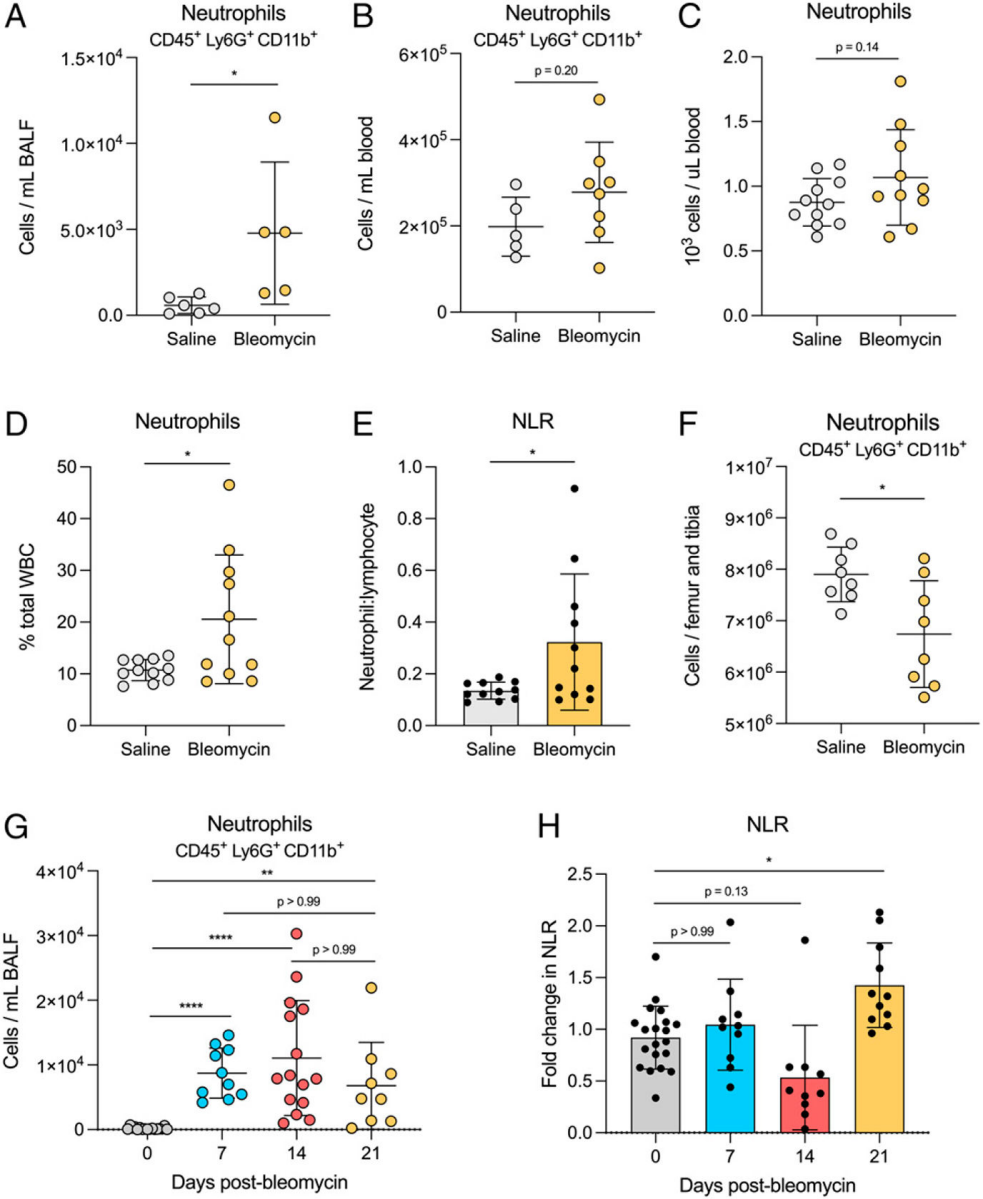
Neutrophil distribution differs across tissues in fibrotic mice. (**A** and **B**) Cells were collected from BALF and blood and stained for neutrophil markers CD45, CD11b, and Ly6G. Neutrophils were quantified using flow cytometry. For A, *n* = 5 or 6 mice per group. For B, *n* = 5–8 mice per group. Data are representative of two independent experiments. (**C**–**E**) Whole blood was collected in EDTA tubes for CBC. Neutrophil quantification is represented as absolute number and percentage of total WBCs. NLR was calculated by dividing the absolute neutrophil count by absolute lymphocyte count. *n* = 10 or 11 mice per group. Data from three combined experiments. (**F**) Total bone marrow cells were collected and stained for flow cytometry as described in A and B. *n* = 8 mice per group. Data from two combined experiments. (**G** and **H**) Cells were collected from BALF and blood on days 0, 7, 14, and 21 and stained as described in A and B. NLR was calculated as described in C–E. For A–F, statistical analysis by unpaired Student *t* test; for G and H, statistical analysis by Kruskal-Wallis test with Dunn’s multiple comparisons test; for all, error bars represent the mean ± SD. *p < 0.05, **p < 0.01, ****p < 0.0001.

We first sought to evaluate the distribution of neutrophils across different tissue compartments after the development of fibrosis in mice. We collected cells from BALF, blood, and bone marrow 21 d after bleomycin or saline treatment and used flow cytometry to quantify neutrophil numbers. We also performed CBC as a secondary method of evaluating neutrophil numbers in blood. Elevated neutrophils were found in the BALF of bleomycin-treated mice compared with saline-treated mice (Fig. 1A). No significant difference in the number of neutrophils in blood was observed by either flow cytometry or CBC (Fig. 1B, 1C); however, we did measure an increase in the frequency of neutrophils per total WBCs and an increased NLR in bleomycin-treated mice (Fig. 1D, 1E). There was no difference in the number of total blood cells (Supplemental Fig. 1A, 1B). Interestingly, in bone marrow, there were decreased neutrophils in bleomycin-treated mice compared with saline-treated mice (Fig. 1F). Bleomycin-induced lung fibrosis proceeds through a multistage disease course starting with inflammation after bleomycin-induced lung injury (days 1–7), fibroproliferation (days 7–14), and active collagen deposition and progression to fibrosis (days 14–21) (20). We evaluated how peripheral neutrophil levels fluctuated over the 21-d time course of bleomycin-induced lung fibrosis. We found that neutrophil numbers in BALF significantly increased over day 0 starting 7 d after bleomycin treatment and remained elevated through 21 d after bleomycin treatment (Fig. 1G). However, we observed elevated NLR only at day 21 compared with day 0 (Fig. 1H).

### Neutrophil trafficking between bone marrow and periphery is altered in fibrosis

**FIGURE 2.**
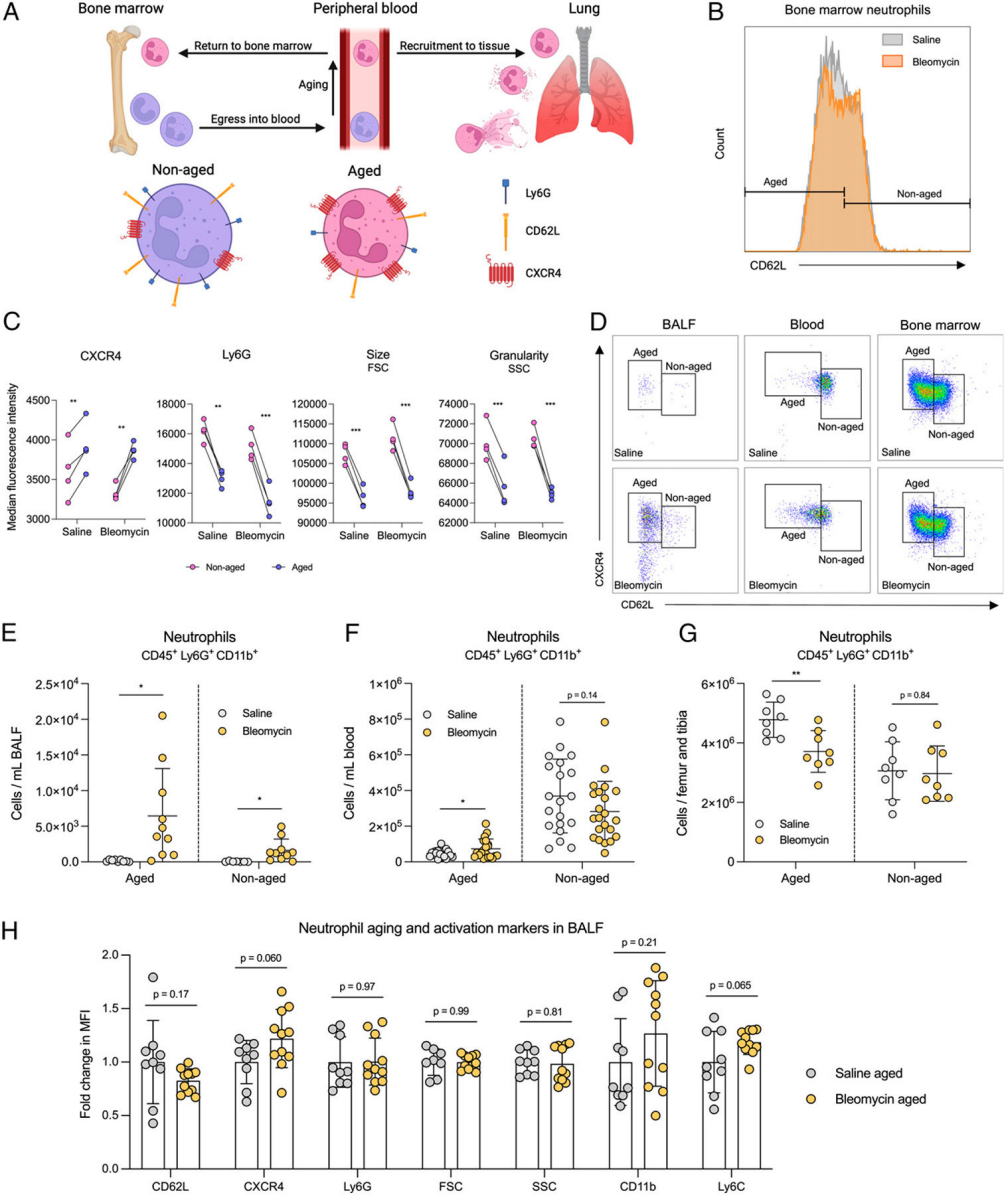
Aged neutrophils accumulate in the periphery of fibrotic mice. (**A**) Neutrophils egress from the bone marrow after development and age while circulating in the blood. Aged neutrophils express low levels of CD62L and Ly6G and high levels of CXCR4. Upon inflammatory stimuli, aged neutrophils are recruited from the circulation into tissue, where they exert effector functions. At steady state (no inflammation), aged neutrophils are recruited back to the bone marrow for clearance. (**B**) Representative histogram showing CD62L MFI on bone marrow neutrophils from saline-treated (gray) and bleomycin-treated (orange) mice. Aged and nonaged neutrophil populations were determined on the basis of CD62L expression. (**C**) MFI of CXCR4, Ly6G, size, and granularity on aged (purple dots) and nonaged (pink dots) bone marrow neutrophils from saline- and bleomycin-treated mice identified as described in B. *n* = 4 mice per group. Data representative of two independent experiments. Statistical analysis by multiple paired *t* tests. (**D**) Representative gating schemes of aged and nonaged neutrophils from BALF, blood, and bone marrow isolated from saline- or bleomycin-treated mice. Neutrophils were pregated on CD45, CD11b, and Ly6G. Neutrophil age was determined on the basis of CD62L and CXCR4 expression. (**E**–**G**) Quantification of aged versus nonaged BALF, blood, and bone marrow neutrophils described in D. Samples were collected as described in Fig. 1. Statistical analysis by unpaired Student *t* test. For E, *n* = 9–11 mice per group; data from two combined experiments. For F, *n* = 21 mice per group; data from four combined experiments. For G, *n* = 8 mice per group; data from two combined experiments. (**H**) MFI of CD62L, CXCR4, Ly6G, FSC, SSC, CD11b, and Ly6C on aged BALF neutrophils from saline- and bleomycin-treated mice identified in E. Statistical analysis by multiple unpaired Student *t* tests; *n* = 9–11 mice per group; data from two combined experiments. For E–H, error bars represent the mean ± SD. *p < 0.05, **p < 0.01, ***p < 0.001.

As neutrophils circulate in the blood, they undergo distinct phenotypic and functional changes through a process known as “aging” before returning to the bone marrow for clearance (Fig. 2A) (21). Aged neutrophils are more proinflammatory than nonaged neutrophils (22-24). Analyzing the location of aged neutrophils can be instructive in understanding their homeostasis because, under steady-state conditions, these proinflammatory cells are cleared in the bone marrow without exerting their effector functions in tissues, which protects from tissue injury. Conversely, increased numbers of aged neutrophils in tissue portend ongoing inflammatory damage. Phenotypic changes of aged neutrophils are identifiable by flow cytometry using the markers CD62L, CXCR4, Ly6G, size (as determined by forward scatter [FSC]), and granularity (as determined by side scatter [SSC]) (21, 22, 24, 25). Decreased size and granularity correlate with both increased age and increased activation of neutrophils through their release of granule contents (24-27). To evaluate neutrophil trafficking and homeostasis in fibrosis, we separated aged and nonaged neutrophils via CD62L expression (Fig. 2B). To validate our differentiation of these neutrophil populations, we quantified the mean fluorescence intensity (MFI) of CXCR4, Ly6G, size, and granularity of aged and nonaged neutrophils from fibrotic and nonfibrotic mice. In accordance with previous studies (21, 22, 24, 25), we observed increased CXCR4 expression, decreased Ly6G expression, and reduced cell size and granularity on aged neutrophils from both fibrotic and nonfibrotic mice (Fig. 2C), validating our method of distinguishing neutrophil age in the bleomycin model of fibrosis. We next quantified the numbers of aged neutrophils present in BALF, blood, and bone marrow of bleomycin- or saline-treated mice based on CD62L and CXCR4 (Fig. 2D, Supplemental Fig. 1C). Our results showed a significant increase in aged neutrophils from BALF and blood and a decrease from bone marrow of bleomycin-treated mice compared with saline-treated mice (Fig. 2E-2G), suggesting dysregulated neutrophil homeostasis in fibrosis. Because aged neutrophils have a phenotype similar to that of activated neutrophils, we compared expression of various aging and activation markers on aged neutrophils from saline-treated mice and aged neutrophils from bleomycin-treated mice. In addition to CD62L, CXCR4, Ly6G, FSC, and SSC, we also measured expression of CD11b (a canonical neutrophil activation marker) and Ly6C (a commonly used marker of inflammatory myeloid cells). We observed no significant difference in the levels of any of these markers (Fig. 2H), suggesting that aged neutrophils from saline- and bleomycin-treated mice are not different in their activation phenotypes.

### Aged neutrophils and a low Ly6C-expressing neutrophil population are persistently elevated after bleomycin treatment

**FIGURE 3.**
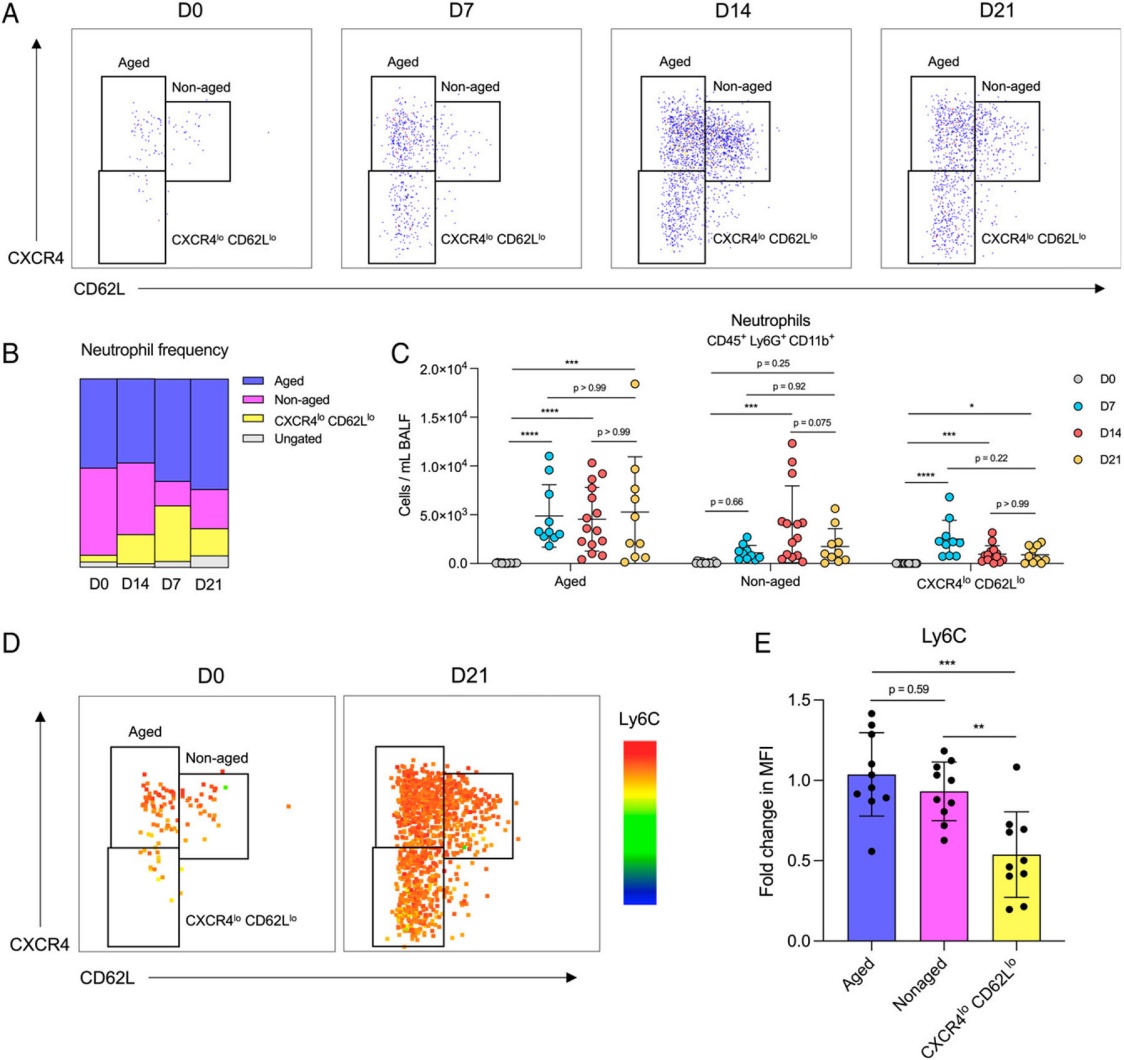
Bleomycin treatment has a sustained effect on the composition of neutrophil populations in BALF. (**A**) Representative gating strategies of BALF neutrophil populations isolated 0, 7, 14, and 21 d after bleomycin treatment. Neutrophils were gated on CD45, CD11b, and Ly6G. (**B**) Frequency of identified neutrophil populations from mice described in A. Data show the means of two combined experiments. (**C**) Numbers of aged, nonaged, and CXCR4^lo^ CD62L^lo^ neutrophils identified in A. *n* = 10–15 mice per group; data from two combined experiments. Statistical analysis by Kruskal-Wallis test with Dunn’s multiple comparisons (aged and CXCR4^lo^ CD62L^lo^) or one-way ANOVA with Tukey’s multiple comparisons (nonaged); error bars represent the mean ± SD. (**D**) Heatmap of Ly6C expression on neutrophils 0 and 21 d after bleomycin. Representative of two independent experiments. (**E**) Quantification of Ly6C expression on aged, nonaged, and CXCR4^lo^ CD62L^lo^ neutrophils from day 21 bleomycin-treated mice. Data from two combined experiments; *n* = 10 mice per group. Statistical analysis by one-way ANOVA with Tukey’s multiple comparisons; error bars represent the mean ± SD. *p < 0.05, ***p < 0.001, ****p < 0.0001.

We next evaluated how the number of aged BALF neutrophils changed over the fibrotic disease time course. We gated aged and nonaged BALF neutrophils on CD62L and CXCR4 as described in Fig. 2 and, in addition, observed the appearance of a CXCR4^lo^ CD62L^lo^ neutrophil population on day 7 that did not resemble aged or nonaged neutrophils (Fig. 3A, 3B). Like the trends in total BALF neutrophils, we quantified an increase in both aged and CXCR4^lo^ CD62L^lo^ neutrophils on day 7 that persisted through day 21 after bleomycin treatment (Fig. 3C). In addition to lower expression of CXCR4 and CD62L, we also determined that the population of CXCR4^lo^ CD62L^lo^ neutrophils had decreased expression of Ly6C (Fig. 3D, 3E), suggesting a decreased inflammatory or activation state.

### CXCL12-CXCR4 and CXCL1/2-CXCR2 signaling axes directing neutrophil migration are disrupted in fibrosis

**FIGURE 4.**
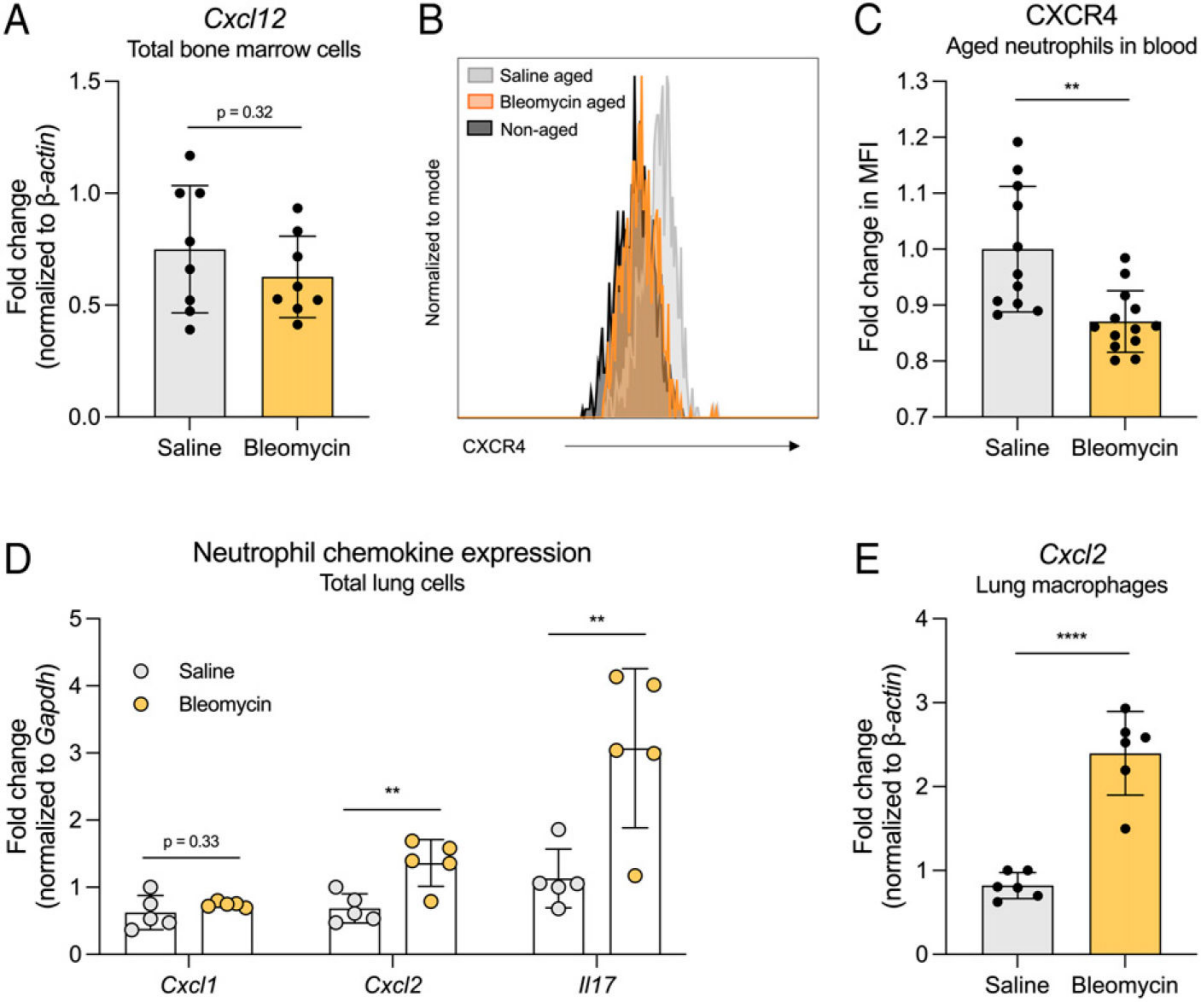
Neutrophil recruitment to and retention within peripheral tissues is likely mediated by decreased neutrophil CXCR4 expression and increased Cxcl2 expression by lung cells in fibrosis. (**A**) Cxcl12 expression by total bone marrow cells measured by RT quantitative PCR (RT-qPCR). Bone marrow cells were flushed from one femur and tibia of each mouse. *n* = 8 mice per group. Data from two combined experiments. (**B**) Representative histogram of CXCR4 MFI on nonaged (black) and aged neutrophils from saline-treated (gray) and bleomycin-treated (orange) mice. Aged neutrophils were identified as described in Fig. 2. (**C**) Quantification of CXCR4 MFI on aged blood neutrophils from B. *n* = 11–13 mice per group. Data from two combined experiments. (**D**) Expression of Cxcl1, Cxcl2, and Il17 by total lung cells collected from fibrotic and nonfibrotic mice. Cells were isolated via collagenase digest, and gene expression was measured via RT-qPCR. *n* = 5 mice per group. Data representative of two independent experiments. (**E**) Expression of Cxcl2 by total lung macrophages collected from fibrotic and nonfibrotic mice. Macrophages were isolated via collagenase digest and adherence purification, and gene expression was measured via RT-qPCR. *n* = 2 or 3 mice per group. Data from two combined experiments. For all figures, statistical analysis by unpaired Student *t* test; error bars represent the mean ± SD. **p < 0.01, ****p < 0.0001.

One mode of regulating neutrophil trafficking between the bone marrow and the blood is through the CXCL12-CXCR4 signaling axis. CXCL12 produced by bone marrow stromal cells signals through its receptor CXCR4 expressed at high levels on aged neutrophils, promoting migration of neutrophils back to the bone marrow for clearance (22, 25, 28). Thus, we first measured *Cxcl12* expression by total bone marrow cells and found no significant difference between bleomycin- and saline-treated mice (Fig. 4A). We next measured the levels of CXCR4 expressed on aged neutrophils in blood (Fig. 4B). We observed a decrease in CXCR4 MFI on neutrophils from bleomycin-treated mice compared with saline-treated mice, suggesting that aged blood neutrophils in fibrotic mice are less sensitive to CXCL12 signaling from the bone marrow, which may limit their clearance (Fig. 4C). When considering neutrophil transmigration through the vascular endothelium and into inflamed tissues, many studies have shown an essential role of platelets for neutrophils to make firm contact with endothelial cells (29-32). Interestingly, we observed increased numbers of platelets in bleomycin-treated mice compared with saline-treated mice, as measured by CBC (Supplemental Fig. 1D). Last, we interrogated the production of CXCL1/2 chemokines, which are produced under inflammatory conditions and are responsible for recruiting neutrophils to sites of inflammation. We isolated total lung cells from fibrotic and nonfibrotic mice via collagenase digest and measured the expression of *Cxcl1* and *Cxcl2*, as well as *Il17*, which is known to upregulate CXCL1/2 expression (33), by RT-qPCR. Although we measured no change in *Cxcl1* expression, we quantified increased *Cxcl2* and *Il17* gene expression by lung cells from bleomycin-treated mice compared with saline-treated mice (Fig. 4D). Macrophages produce CXCL2 and are important for directing neutrophils to sites of inflammation within tissue (34, 35); therefore, we quantified *Cxcl2* expression by lung macrophages from fibrotic mice and nonfibrotic mice. Isolated lung macrophages from bleomycin-treated mice expressed increased *Cxcl2* mRNA compared with lung macrophages from saline-treated mice (Fig. 4E), suggesting that tissue-resident macrophages may play a role in neutrophil recruitment to the airspaces in fibrosis.

### Neutrophils in fibrosis are primed for elevated granule and NET release

**FIGURE 5.**
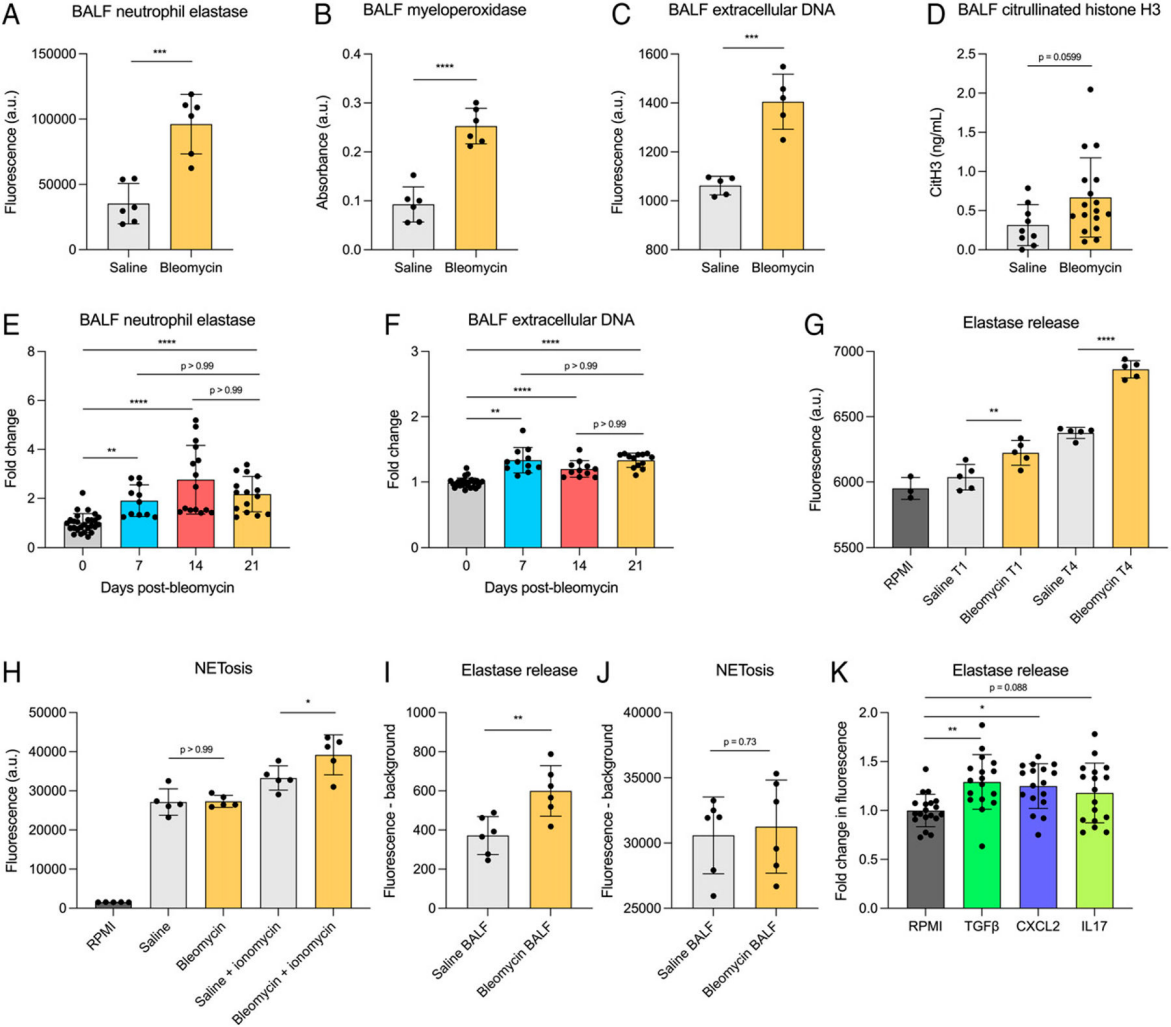
Neutrophils in fibrotic mice release increased levels of NE, MPO, and NETs. (**A**–**D**) NE (A) MPO (B), exDNA (C), and CitH3 (D) measured in cell-free BALF collected from saline- or bleomycin-treated mice. *n* = 6 mice per group (A–C) or 9–17 mice per group (D). Data representative of two independent experiments (A–C) or two combined experiments (D). Statistical analysis by unpaired Student *t* test (A–C) or Mann-Whitney *U* test (D). (**E**, **F**) NE (E) and exDNA (F) measured in cell-free BALF from mice 0, 7, 14, and 21 d after bleomycin treatment. *n* = 11–27 mice per group. Data from two combined experiments. Statistical analysis by one-way ANOVA with Tukey’s multiple comparisons. (**G**) Unstimulated ex vivo NE release by bone marrow neutrophils collected from saline- or bleomycin-treated mice. Supernatant was collected for analysis from ex vivo cultures 1 h and 4 h after plating. *n* = 2 or 3 mice per group, dots represent technical replicates. Data representative of two independent experiments. Statistical analysis by one-way ANOVA with Sidak’s multiple comparisons test. (**H**) Ex vivo NETosis by bone marrow neutrophils collected from saline- or bleomycin-treated mice. NETosis was measured after 5 h in both unstimulated and 5 μM ionomycin-treated conditions. Data representative of two independent experiments. Statistical analysis by one-way ANOVA with Sidak’s multiple comparisons test. (**I**, **J**) Ex vivo NE release (G) and NETosis (H) were measured as described in E and F. Bone marrow neutrophils were isolated from naïve mice and treated with saline or bleomycin BALF collected in plain RPMI. NE release was measured after 4 h, and NETosis was measured after 5 h. Background fluorescence in cell-free BALF was subtracted from samples containing CM and cells. Data representative of two independent experiments. Statistical analysis by unpaired Student *t* test. (**K**) Ex vivo NE release by naive bone marrow neutrophils stimulated with TGF-β, CXCL2, and IL-17 for 4 h. Data from three combined experiments. Statistical analysis by one-way ANOVA with Dunnett’s multiple comparisons test. For all experiments, error bars represent the mean ± SD. *p < 0.05, **p < 0.01, ***p < 0.001, ****p < 0.0001.

We next sought to understand the implications of elevated neutrophil numbers present in fibrotic lungs. We measured the levels of NE, MPO, exDNA, and CitH3 as markers of neutrophil activation via degranulation and NETosis in cell-free BALF from fibrotic and nonfibrotic mice. We found that both NE and MPO were increased in BALF from bleomycin-treated mice relative to saline-treated mice (Fig. 5A, 5B), suggesting that neutrophils in fibrosis release more granules. There was also significantly higher exDNA and a strong trend toward increased CitH3 in BALF from bleomycin-treated mice compared with saline-treated mice (Fig. 5C, 5D), indicating higher levels of NETosis. We wanted to understand how the levels of neutrophil products present in BALF changed throughout the bleomycin-induced fibrosis time course; therefore, we quantified NE and exDNA in BALF 0, 7, 14, and 21 d after bleomycin treatment. We found that the levels of both NE and exDNA increased starting on day 7 and remained elevated through day 21 (Fig. 5E, 5F). To determine whether this was due to differences in neutrophil function rather than just an increase in neutrophil numbers in fibrotic mice, we isolated bone marrow neutrophils from day 21 bleomycin- and saline-treated mice and performed ex vivo functional assays to quantify NE release and NETosis by an equal number of cells. NE release was measured in unstimulated cells after 1 h and 4 h of ex vivo culture, and, at both time points, NE was increased in the supernatant of neutrophils from bleomycin-treated mice compared with saline-treated mice (Fig. 5G). NETosis was measured in both unstimulated neutrophils and neutrophils stimulated with ionomycin for 5 h. Unstimulated neutrophils showed no difference in NETosis; however, ionomycin-stimulated neutrophils from bleomycin-treated mice had increased NETosis compared with those from saline-treated mice (Fig. 5H).

We next wanted to understand the role of soluble mediators produced in the fibrotic lung in regulating neutrophil degranulation and NETosis. To evaluate this, we treated naive neutrophils with BALF from bleomycin- and saline-treated mice (herein termed “bleomycin BALF” and “saline BALF”) and measured NE release and NETosis after 4 h and 5 h, respectively. We found that treatment with bleomycin BALF significantly increased NE release by naive neutrophils compared with treatment with saline BALF (Fig. 5I) Interestingly, we observed no difference in NETosis when treating with bleomycin BALF compared with saline BALF (Fig. 5J). We hypothesized that cytokines known to be upregulated in fibrosis were mediating the increased NE release measured when treating naive neutrophils with bleomycin BALF relative to saline BALF. On the basis of our data in Fig. 4D demonstrating that *Cxcl2* and *Il17* are upregulated in bleomycin BALF, as well as previous studies showing elevated TGF-β in fibrotic lungs (36, 37), we treated neutrophils with re-combinant CXCL2, TGF-β, and IL-17 and measured NE release after 4 h of ex vivo culture. We found that both CXCL2 and TGF-β significantly increased levels of NE present in supernatant compared with the plain RPMI control, whereas there was a trending increase in NE release that did not reach statistical significance when neutrophils were treated with IL-17 (Fig. 5K).

### Increased viability of airspace neutrophils in fibrosis

We collected neutrophils from BALF of fibrotic and nonfibrotic mice and stained them with Apotracker Green and Zombie Violet to measure cell viability and quantify the ratios of live, early apoptotic, late apoptotic, and secondary necrotic cells (Supplemental Fig. 2A, 2B). Heat-shocked BALF cells were used as a positive control for cell death. Strikingly, we observed a significantly greater proportion of live neutrophils from bleomycin-treated mice than from saline-treated mice, suggesting that apoptosis is inhibited in neutrophils from fibrotic mice (Supplemental Fig. 2C). Although the frequencies of early apoptotic neutrophils were similar between fibrotic and nonfibrotic mice, we observed a decrease in the frequency of neutrophils in late apoptosis and an increase in the frequency of neutrophils undergoing secondary necrosis from bleomycin BALF compared with saline BALF (Supplemental Fig. 2D-2F).

### Varied response to bleomycin shows that poor disease outcome is associated with increased airspace neutrophils

**FIGURE 6.**
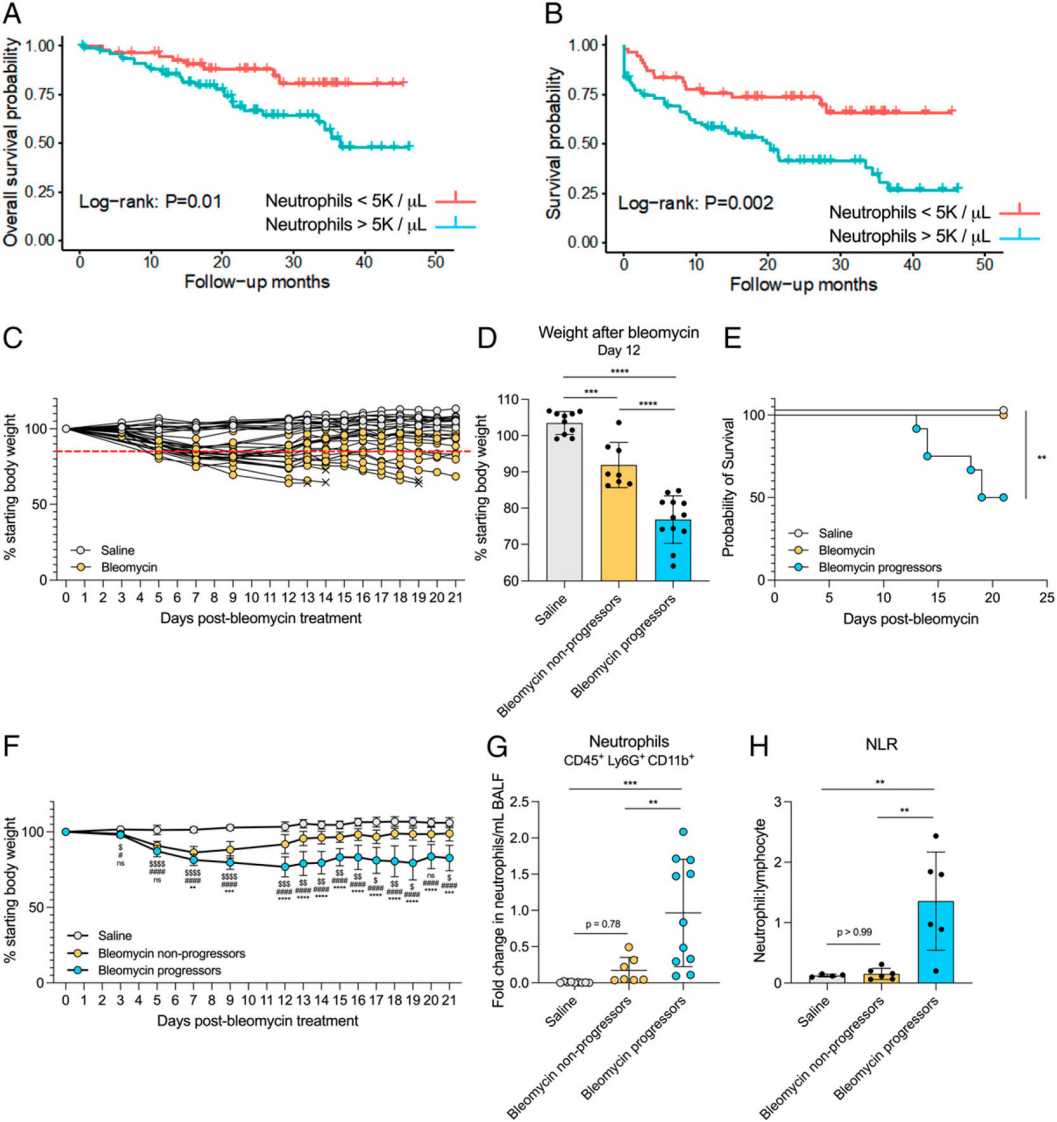
Increased neutrophils are associated with poor outcomes in fibrotic mice. (**A** and **B**) Kaplan-Meier estimates of time to death (A) or first respiratory hospitalization (B) in patients with IPF. (**C**) Percentage starting body weight of individual saline- or bleomycin-treated mice over 21 d. × indicates euthanasia. Red line denotes 15% weight loss threshold. (**D**) Percentage starting body weight of saline- and bleomycin-treated mice 12 d after treatment. Bleomycin-treated mice were divided into two groups based on weight loss: Bleomycin nonprogressors lost <15% and bleomycin progressors lost >15% of their starting body weight by day 12. *n* = 8–12 mice per group. Data from two combined experiments. Statistical analysis by one-way ANOVA with Tukey’s multiple comparisons. (**E**) Survival of mice in saline, bleomycin nonprogressor, and bleomycin progressor groups over 21 d. Mice were euthanized when moribund. *n* = 8–12 mice per group. Data from two combined experiments. Statistical analysis by log-rank test. (**F**) Percentage starting body weight of mice in saline, bleomycin nonprogressor, and bleomycin progressor groups over 21 d. *n* = 8–12 mice per group. Data from two combined experiments. Statistical analysis by one-way ANOVA with Sidak’s multiple comparisons between bleomycin nonprogressors and bleomycin progressors (*), bleomycin nonprogressors and saline ($), and bleomycin progressors and saline (#). (**G**) BALF neutrophils from mice in groups described in D. Neutrophils were quantified via flow cytometry. *n* = 7–11 mice per group. Data from two combined experiments expressed as fold change. Statistical analysis by one-way ANOVA with Tukey’s multiple comparisons. (**H**) NLR in blood from mice in groups described in D. Cells were quantified via flow cytometry. *n* = 5 or 6 mice per group. Data representative of two individual experiments. Statistical analysis by one-way ANOVA with Tukey’s multiple comparisons. For D, F–H, error bars represent the mean ± SD. **p < 0.01, ***p < 0.001, ****p < 0.0001.

To explore the hypothesis that increased peripheral blood neutrophils in patients with IPF are associated with poor clinical outcomes, we retrospectively analyzed survival in a cohort of patients with IPF being followed in the ILD Clinic at the University of Michigan. Demographic and clinical characteristics of the patient cohort are described in Table I. Patients with baseline peripheral neutrophil counts >5000/μl have a significantly increased hazard for all-cause mortality (Fig. 6A). Patients with baseline peripheral neutrophil counts >5000/μl also demonstrated an increased risk of mortality or decreased time to first respiratory hospitalization compared with patients with lower neutrophils counts (<5000/μl) (Fig. 6B). Multivariate analysis using a Cox proportional hazards model demonstrated an increased hazard for all-cause mortality in patients with an elevated neutrophil count (continuous variable), and significance was maintained after adjusting for age, sex, smoking status, and baseline FVC (HR, 1.14; 95% confidence interval, 1.02–1.24; *p* = 0.01). In multivariate analysis for time to death or first respiratory hospitalization, an elevated neutrophil count was also associated with an increased risk of both, which maintained statistical significance after adjusting for age, sex, smoking status, and baseline FVC (HR, 1.10; 95% confidence interval, 1.01–1.18; *p* = 0.03). In conclusion, we found that increased peripheral blood neutrophils were significantly associated with an increased risk of mortality and respiratory hospitalization in patients with IPF, supporting findings from other studies (Fig. 6A, 6B) (3-5).

To experimentally evaluate the association between neutrophil numbers and disease outcome, we took advantage of the inherent variability of the bleomycin model of fibrosis, wherein outcome is not consistent between all mice in a single experiment. We treated mice with saline and bleomycin and monitored their survival and weight loss over 21 d to understand the natural history of disease progression, which is not uniform in all mice (Fig. 6C). We found that the bleomycin-treated mice could be divided into two distinct groups based on percentage starting body weight 12 d after bleomycin treatment: Mice that lost greater than 15% starting body weight were termed “bleomycin progressors,” and mice that lost less than 15% starting body weight were termed “bleomycin nonprogressors” (Fig. 6C, 6D). Throughout the remainder of the experiment, mice that became moribund were euthanized. Criteria for euthanasia are described in Methods. A “poor outcome” was defined as death/euthanasia or weight loss greater than 15% starting body weight 21 d after bleomycin treatment. Bleomycin progressors had significantly increased mortality and weight loss compared with bleomycin nonprogressors days 12–21 after bleomycin treatment (Fig. 6E, 6F) demonstrating that weight loss at day 12 after bleomycin treatment is predictive of a poor outcome. BALF, lung tissue, and blood were collected from all mice at the time of euthanasia. We next quantified the number of neutrophils present in BALF and peripheral blood of saline, bleomycin nonprogressor, and bleomycin progressor groups. Notably, we observed no difference in BALF neutrophils in the saline group compared with the bleomycin nonprogressor group; however, there was a striking increase in neutrophils in the bleomycin progressor group compared with both saline and bleomycin nonprogressor groups (Fig. 6G). Although we observed no significant difference in the absolute number of blood neutrophils between the three groups, there was a significant increase in the NLR in the bleomycin progressor group compared with both the saline and bleomycin nonprogressor groups (Supplemental Fig. 3A, Fig. 6H).

### Poor disease outcome is associated with increased neutrophil activation and NETosis markers

**FIGURE 7.**
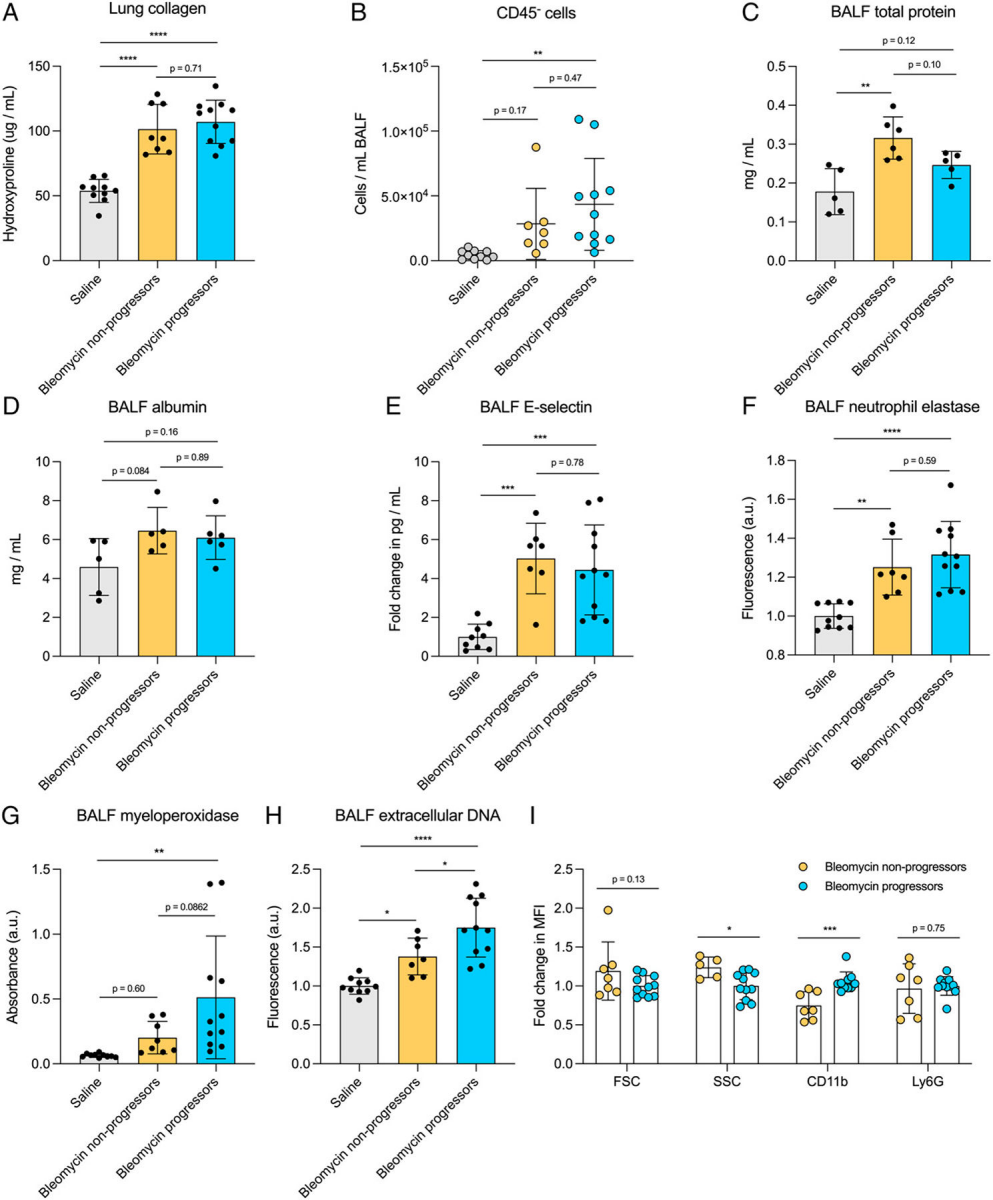
No difference in lung collagen levels but increased markers of BALF neutrophil activation and NETosis in fibrotic mice with poor outcomes. (**A**) Lung collagen levels measured via hydroxyproline assay in mice described in Fig. 6. *n* = 8–12 mice per group. Data from two combined experiments. Statistical analysis by one-way ANOVA with Tukey’s multiple comparisons. (**B**) CD45^−^ cells in BALF measured via flow cytometry from mice described in Fig. 6. *n* = 8–12 mice per group. Data from two combined experiments. Statistical analysis by one-way ANOVA with Tukey’s multiple comparisons. (**C**–**E**) Total protein (C), albumin (D), and E-selectin in BALF from mice described in Fig. 6. *n* = 8–12 mice per group. Data from two combined experiments. Statistical analysis by one-way ANOVA with Tukey’s multiple comparisons. (**F**–**H**) NE (F), MPO (G), and exDNA (H) levels in cell-free BALF collected from mice described in Fig. 6. *n* = 7–10 mice per group. Data from two combined experiments. Statistical analysis by one-way ANOVA with Tukey’s multiple comparisons. (**I**) MFI of FSC, SSC, CD11b, and Ly6G on neutrophils from bleomycin nonprogressor and bleomycin progressor mice described in Fig. 6. *n* = 7–10 mice per group. Data from two combined experiments. Statistical analysis by multiple unpaired Student *t* tests. For all figures, error bars represent the mean ± SD. *p < 0.05, **p < 0.01, ***p < 0.001, ****p < 0.0001.

To determine if the increased neutrophils present in bleomycin progressor mice were associated with increased levels of lung fibrosis, we measured collagen deposition in saline-treated, bleomycin nonprogressor, and bleomycin progressor mice via hydroxyproline assay. (Note that six of the progressor mice were euthanized before day 21, as noted in Fig. 6, and collagen levels represent days 13, 14, 18, and 19 for these mice.) We observed no difference in lung collagen between the bleomycin nonprogressor and progressor groups (Fig. 7A); however, due to survivor bias, it is possible that had all the mice in the bleomycin progressor group survived, they would have had increased hydroxyproline levels. We also cultured normal fibroblasts in media conditioned with supernatant from saline- or bleomycin-isolated neutrophil culture (herein referred to as “saline CM” and “bleomycin CM”) to evaluate profibrotic responses. There was no difference in fibroblast proliferation or expression of fibrosis-associated genes *Col1a1* and *Fn1* between fibroblasts treated with saline CM and bleomycin CM (Supplemental Fig. 3B-3D analyzed using primers in Table II). We next evaluated if the increased neutrophils and morbidity in the bleomycin progressor group were associated with increased lung injury. We first quantified the numbers of CD45^−^ cells present in BALF as a representation of dead or dying epithelial/endothelial cells sloughed off into the airspaces and observed no difference between bleomycin nonprogressor and bleomycin progressor mice (Fig. 7B). We next evaluated the levels of total protein, albumin, and E-selectin present in BALF as a measurement of lung leak and endothelial cell injury. Again, we observed no difference in total protein, albumin, and E-selectin between bleomycin nonprogressor and bleomycin progressor mice (Fig. 7C-7E). Taken together, these data suggest that increased neutrophils present in bleomycin progressor mice do not drive increased lung injury.

We next measured NE, MPO, and exDNA to evaluate neutrophil function in bleomycin nonprogressors compared with bleomycin progressors. We found no significant difference in BALF NE and a trending increase in MPO between the two groups (Fig. 7F, 7G); however, there was a significant increase in BALF exDNA in the bleomycin progressor group compared with bleomycin nonprogressors (Fig. 7H), suggesting that neutrophils in fibrotic mice that have poor outcomes release more NETs.

There was no significant difference in plasma levels of NE or exDNA between bleomycin progressors and bleomycin nonprogressors (Supplemental Fig. 3E, 3F). We also evaluated neutrophil activation via MFI of FSC, SSC, and CD11b. Although there was no significant difference in FSC, neutrophils from bleomycin progressor mice had decreased SSC and increased CD11b expression relative to neutrophils from bleomycin nonprogressor mice (Fig. 7I), further suggesting a more activated phenotype. To verify that the difference in activation state was not due to an increase in aged neutrophils in progressor mice, we also measured Ly6G expression and observed no difference compared with bleomycin nonprogressor mice (Fig. 7I).

### Anti-Ly6G (clone 1A8) does not effectively deplete neutrophils in bleomycin-treated mice

We next wanted to test the role of neutrophils in the fibrotic phase of the bleomycin time course, so we treated fibrotic and nonfibrotic mice with anti-Ly6G Abs (clone 1A8) every 48 h starting at day 12 after bleomycin. At 21 d after bleomycin, we collected blood and BALF and quantified neutrophil numbers by gating on CD11b and Ly6G or CD11b and SSC. In blood, gating by CD11b and Ly6G resulted in an apparent decrease in neutrophils in bleomycin-treated mice receiving anti-Ly6G (bleomycin + anti-Ly6G) compared with bleomycin-treated mice receiving PBS (bleomycin + PBS) (Supplemental Fig. 4A, 4B). However, gating by CD11b and SSC revealed a population of cells resembling neutrophils that was present in both bleomycin + PBS and bleomycin + anti-Ly6G-treated mice (Supplemental Fig. 4A, 4C). This cell population was positive for Ly6G in bleomycin + PBS-treated mice and negative for Ly6G in bleomycin + anti-Ly6G-treated mice (Supplemental Fig. 4A, comparing top and bottom panels), suggesting that our neutrophil depletion method was not successful and that pretreatment with anti--Ly6G had masked extracellular Ags, making them undetectable via flow cytometry. We did not observe significant depletion of neutrophils in bleomycin BALF using either CD11b and Ly6G or CD11b and SSC for identification (Supplemental Fig. 4D, 4E).

## DISCUSSION

Increased neutrophils are associated with poor outcomes in IPF (2-6); however, their precise role in the pathogenesis of lung fibrosis is undetermined. Furthermore, although previous studies showed that neutrophil products such as NE and NETs promote myofibroblast differentiation, activation, and collagen production (13-17), it is unknown how alterations in neutrophil homeostasis or function may further contribute to fibrotic disease progression. In the present study, we show that neutrophils in fibrosis preferentially traffic to the airspaces and appear to be primed for activation, releasing increased levels of NE and NETs.

We hypothesized that, like in patients with IPF, we would observe elevated numbers of neutrophils in BALF and blood of mice with established fibrosis (day 21 after bleomycin treatment). Indeed, we did observe increased neutrophils in BALF and an increase in NLR in the blood of fibrotic mice compared with nonfibrotic mice. Interestingly, we also measured decreased numbers of neutrophils in the bone marrow of fibrotic mice. On the basis of these findings, we next hypothesized that neutrophils in fibrotic mice were egressing from the bone marrow into the circulation and actively being recruited into the lung, avoiding clearance in the bone marrow. To test this, we measured the numbers of aged and nonaged neutrophils present in BALF, blood, and bone marrow. After neutrophils age, they either traffic back to the bone marrow or are recruited into peripheral tissues by proinflammatory signals. We postulated that if neutrophil trafficking were dysregulated in fibrosis and neutrophils were erroneously being recruited into the lungs, we would observe increased aged neutrophils in samples from peripheral tissues and decreased aged neutrophils in bone marrow. Accordingly, the numbers of aged neutrophils in fibrotic mice were increased in BALF and blood and decreased in bone marrow compared with nonfibrotic mice. These results are consistent with dysregulated neutrophil homeostasis in fibrotic mice, characterized by accumulation of aged neutrophils in the periphery with a failure of those aged cells to return to the bone marrow for clearance. Neutrophil trafficking between the bone marrow and the periphery is important for maintenance of the bone marrow niche and production of various leukocyte populations (25, 38, 39); therefore, altered neutrophil trafficking in fibrosis may have farther-reaching long-term effects on bone marrow homeostasis. Future studies will seek to understand the implications of altered neutrophil trafficking on the production of leukocyte populations in the bone marrow.

Interestingly, we also noted the appearance of a CD11b^+^ Ly6G^+^ CXCR4^lo^ CD62L^lo^ Ly6C^lo^ population of neutrophils in BALF starting at day 7 and persisting through day 21 after bleomycin. Other studies have identified CD11b^+^ Ly6G^+^ Ly6C^lo^ cells as polymorphonuclear myeloid-derived suppressor cells (PMN-MDSCs) (40, 41); therefore it is possible that the population of CXCR4^lo^ CD62L^lo^ Ly6C^lo^ cells in our bleomycin model are PMN-MDSCs, although conclusive identification will require measurements of their suppressor function. These cells are thought to arise after persistent immune activation and have been described in various chronic lung diseases (42-47). Monocytic MDSCs have already been described in pulmonary fibrosis (48); however, the role of PMN-MDSCs has not been evaluated and is worth further study.

To better understand how fibrosis alters neutrophil chemo-taxis and homeostasis, we interrogated the CXCL12-CXCR4 and CXCL1/2-CXCR2 signaling axes. CXCL12 is produced by bone marrow stromal cells and signals through CXCR4, which is upregulated on aged neutrophils and recruits them back to the bone marrow for clearance (22, 25, 28). Although there was no difference in *Cxcl12* expression by bone marrow cells, suggesting that the bone marrow retains neutrophil-recruiting capacity, CXCR4 was significantly downregulated on aged blood neutrophils from fibrotic mice. Thus, neutrophils in fibrosis are likely less sensitive to CXCL12 signaling and do not robustly return to the bone marrow for clearance. Conversely, CXCL1/2 is upregulated in peripheral tissues during inflammation and rapidly drives neutrophil recruitment via CXCR2 signaling. Interestingly, we found *Cxcl2* to be upregulated in total lung cells. CXCL2 is highly expressed by tissue-resident macrophages during inflammation (34, 35, 49); therefore, we measured lung macrophage *Cxcl2* expression and found that macrophages from fibrotic mice expressed significantly higher levels of the chemokine than macrophages from nonfibrotic mice. Studies have identified macrophage subsets contributing to the development of fibrosis (50, 51), and it is possible that these macrophage populations drive the increased *Cxcl2* expression and neutrophil recruitment.

We next evaluated whether neutrophils in fibrotic mice were more activated than in nonfibrotic mice. In support of this, we measured increased levels of soluble, cell-free NE, MPO, exDNA, and CitH3, markers of neutrophil activation via degranulation and NETosis. When assayed ex vivo, we found that neutrophils from fibrotic mice release more NE and NETs, indicating that these neutrophils are primed for activation compared with neutrophils from nonfibrotic mice. To determine the role of soluble mediators within the fibrotic lung driving increased neutrophil activation, we treated naive neutrophils with BALF from fibrotic and nonfibrotic mice and measured NE release and NETosis ex vivo. Interestingly, treatment with BALF from fibrotic mice increased NE release but not NETosis compared with BALF from nonfibrotic mice. When we take this in conjunction with results from Fig. 5H, showing that neutrophils from fibrotic mice have an intrinsic ability to undergo NETosis when maximally stimulated, we interpret these results to suggest that neutrophils in fibrotic mice may not be triggered by soluble mediators alone in the BALF. Combined, these results identify the neutrophils of fibrotic mice to have a more activated phenotype characterized by increased release of NE and intrinsic NETosis. Although our study has not identified the reasons for the intrinsically greater NETosis phenotype in fibrotic neutrophils, it may reflect the larger proportion of aged neutrophils in the periphery and lung of fibrotic mice and may be consistent with our finding of increased necrosis in BALF neutrophils.

We previously published that neutrophil inflammatory functions are impaired in the setting of methicillin-resistant *Staphylococcus aureus* lung infection during fibrosis (52). These findings are seemingly at odds with the present study and indicate that the regulation of neutrophil function is multifaceted and context dependent, differing during infection/inflammation versus fibrosis at baseline; however, the mechanisms driving these differential responses are unknown. One possibility is that the different soluble mediators that neutrophils encounter during inflammation versus homeostasis regulate their altered function in fibrosis. We have previously shown that CXCL2 production is inhibited in fibrotic mice during infection (52), and, in the present study, we demonstrate that CXCL2 stimulation increases NE release by naive neutrophils. Therefore, CXCL2 as well as other soluble mediators produced in the fibrotic lung before and after infection should be broadly evaluated and interrogated for their ability to drive or inhibit neutrophil antimicrobial responses. Future studies will aim to unravel the mechanisms dictating differential neutrophil responses in fibrosis at baseline versus during infection.

Finally, we wanted to understand the role neutrophils play in fibrotic disease outcome. On the basis of weight loss 12 d after bleomycin treatment, we found that mice could be divided into “progressor” (>15% weight loss) and “nonprogressor” (<15% weight loss) groups. Bleomycin progressors had significantly increased mortality, which was associated with elevated neutrophil numbers in BALF and increased NLR in blood compared with bleomycin nonprogressors. This is consistent with the data in Fig. 1 demonstrating the variability in neutrophil numbers in fibrotic mice and showing that not all fibrotic mice develop increased levels of peripheral neutrophils but demonstrate those that do show these increases have worse outcomes. We hypothesized that increased mortality and neutrophils would be associated with increased lung collagen, but, surprisingly, there was no difference in hydroxyproline levels between progressors and nonprogressors, although this result may be impacted by survivor bias. Interestingly, however, there was increased exDNA in BALF from progressors versus nonprogressors, suggesting enhanced NETosis in progressor mice. It is unlikely that the increased DNA came from injured epithelial or endothelial cells, because there was no difference in markers of lung injury or leak between bleomycin progressors and bleomycin nonprogressors. In addition, neutrophils in bleomycin progressor mice exhibited a more activated phenotype, as measured by decreased granularity and increased CD11b expression compared with neutrophils from bleomycin nonprogressor mice. Taken together, these data strongly suggest that the mice which have the worst outcomes after bleomycin experience increased neutrophil activation and NETosis, which may promote impaired gas exchange and respiratory function. Future studies will evaluate the role of neutrophils driving disease exacerbation via impairment of normal lung function.

To summarize the present findings, we have identified alterations to the trafficking and function of neutrophils in fibrosis that likely contribute to disease progression and poor outcomes. In addition, aged, proinflammatory neutrophils are elevated in the blood and BALF of fibrotic mice. Neutrophil retention in the periphery and recruitment to the airspaces in fibrosis is likely promoted through downregulation of the CXCL12–CXCR4 axis and upregulation of the CXCL1/2–CXCR2 axis, respectively. We also demonstrated that neutrophils in fibrosis are more activated, releasing increased NE and NETs compared with neutrophils in nonfibrotic mice. Interestingly, fibrotic mice with progressive phenotypes and poor disease outcomes had increased activated neutrophils and elevated levels of exDNA in BALF compared with fibrotic mice with a nonprogressive phenotype, suggesting that increased NETosis is associated with increased morbidity and mortality. Together, the findings described in this study further characterize neutrophil subsets in fibrotic lung disease and may provide information to target neutrophil trafficking or function as a means of preventing disease exacerbation and poor outcomes in fibrosis.

## Supplementary Material

Supplemental data

## Figures and Tables

**TABLE I.** IPF patient cohort characteristics

	All (N = 133)	Neutrophils <5000/μl (n = 54)	Neutrophils >5000/μl (n = 79)
Age, y mean ± SD	69.3 ± 8.7	68.3 ± 8.7	69.9 ± 8.7
Sex, n (%)			
Female	42 (32%)	16 (30%)	26 (33%)
Male	91 (68%)	38 (70%)	53 (67%)
Smoker, n (%)			
Active smoker	5 (4%)	1 (2%)	4 (5%)
Ex-smoker	87 (65%)	33 (61%)	54 (68%)
Nonsmoker	41 (31%)	20 (37%)	21 (27%)
FVC, mean % predicted ± SD	68.5 ± 16.9	71.8 ± 17.3	66.3 ± 16.4
DLCO, mean % predicted ± SD	49.6 ± 17.4	56.5 ± 17.6	44.3 ± 15.5

DLCO, diffusing capacity of the lung for carbon monoxide.

**TABLE II.** Primer and probe sequences

Name	Sequence (5′-3′)	Strand	Modification
*Bactin*	5′-CCGTGAAAAGATGACCCAGATC-3′	Forward	
	5′-CACAGCCTGGATGGCTACGT-3′	Reverse	
	5′-TTTGAGACCTTCAACACCCCA-3′	Probe	5′Fam-3′Tamra
*Cxcl12*	5′-CTCTCTGCTTGCCTCCAAAC-3′	Forward	
	5′-ACTCTCCTCCCTTCCATTGC-3′	Reverse	
	5′-CACCTCTGTAGCCTGACGGACCA-3′	Probe	5′Fam-3′Tamra
*Cxcl1*	5′-GCGCCTATCGCCAATGAG-3′	Forward	
	5′-GCAACACCTTCAAGCTCTGGAT-3′	Reverse	
	5′-TGCCTGCAGACCATGGCTGGG-3′	Probe	5′Fam-3′Tamra
*Cxcl2*	5′-CCTGCCAAGGGTTGACTTCA-3′	Forward	
	5′-CCTTGAGAGTGGCTATGACTTCTG-3′	Reverse	
	5′-CGCCCCCAGGACCCCACTG-3′	Probe	5′Fam-3′Tamra
*Il17*	5′-CCGCAATGAAGACCCTGATAG-3′	Forward	
	5′-GCTTTCCCTCCGCATTGA-3′	Reverse	
	5′-GGGAAGCTCAGTGCCGCCAG-3′	Probe	5′Fam-3′Tamra
*Col1a1*	5′-TGACTGGAAGAGCGGAGAGTACT-3′	Forward	
	5′-GGTCTGACCTGTCTCCATGTTG-3′	Reverse	
	5′-CTGCAACCTGGACGCCATCAAGG-3′	Probe	5′Fam-3′Tamra
*Fn1*	5′-TCGAGCCCTGAGGATGGA-3′	Forward	
	5′-GTGCAAGGCAACCACACTGA-3′	Reverse	
	5′-CTGCAGGGCCTCCAGGCCGG-3′	Probe	5′Fam-3′Tamra

## Abbreviations used in this article:

ACK: ammonium-chloride-potassium
BALF: bronchoalveolar lavage fluid
BCA: bicinchoninic acid
CBC: complete blood count
CitH3: citrullinated histone H3
CM: conditioned media
exDNA: extracellular DNA
FSC: forward scatter
HR: hazard ratio
ILD: interstitial lung disease
IPF: idiopathic pulmonary fibrosis
MFI: mean fluorescence intensity
MPO: myeloperoxidase
NE: neutrophil elastase
NET: neutrophil extracellular trap
NLR: neutrophil-to-lymphocyte ratio
PMN-MDSC: polymorphonuclear myeloid-derived suppressor cell
SSC: side scatter
