# Supplementary material for: Fibrotic Lung Disease Alters Neutrophil Trafficking and Promotes Neutrophil Elastase and Extracellular Trap Release
Source: Immunohorizons. Author manuscript; Available in PMC 2023 Oct 1. (PMC10542701; doi:10.4049/immunohorizons.2200083)
Supplement: Supplemental data [file NIHMS1932051-supplement-Supplemental_data.pdf]

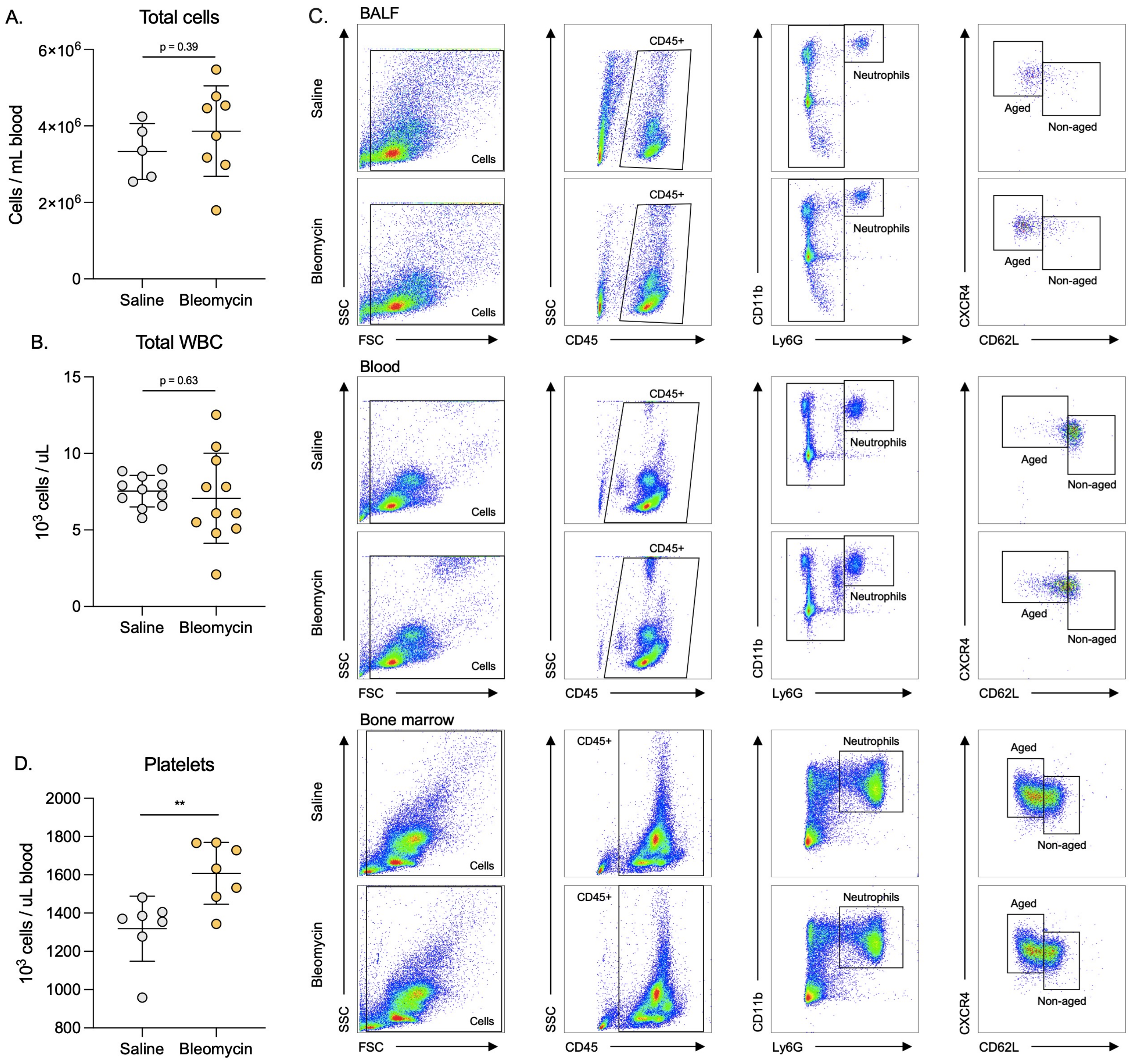

Supplemental Figure 1: No difference in total WBC numbers in fibrotic mice. A. Cells were collected from blood and quantified via flow cytometry. WBCs were gated as all CD45$^+$ cells. N = 5-8 mice per group. Data representative of >2 independent experiments. **B.** Whole blood was collected in EDTA tubes for complete blood count. WBC quantification is represented as absolute number. N = 11 mice per group. Data from 3 combined experiments. **C.** Identification of aged and non-aged neutrophils via flow cytometry from BALF, blood, and bone marrow isolated from saline and bleomycin-treated mice. **D.** Absolute number of platelets as measured by CBC on whole blood from fibrotic and nonfibrotic mice. N = 7 mice per group. Data from 2 combined experiments. For A-B and D: statistical analysis by unpaired Student's t test; error bars represent the means +/- SD.

Downloaded from http://journals.aai.org/immunohorizons/article-supplement/237590/pdf/immunohorizon_2200083_supplemental_1/ by guest on 26 September 2023

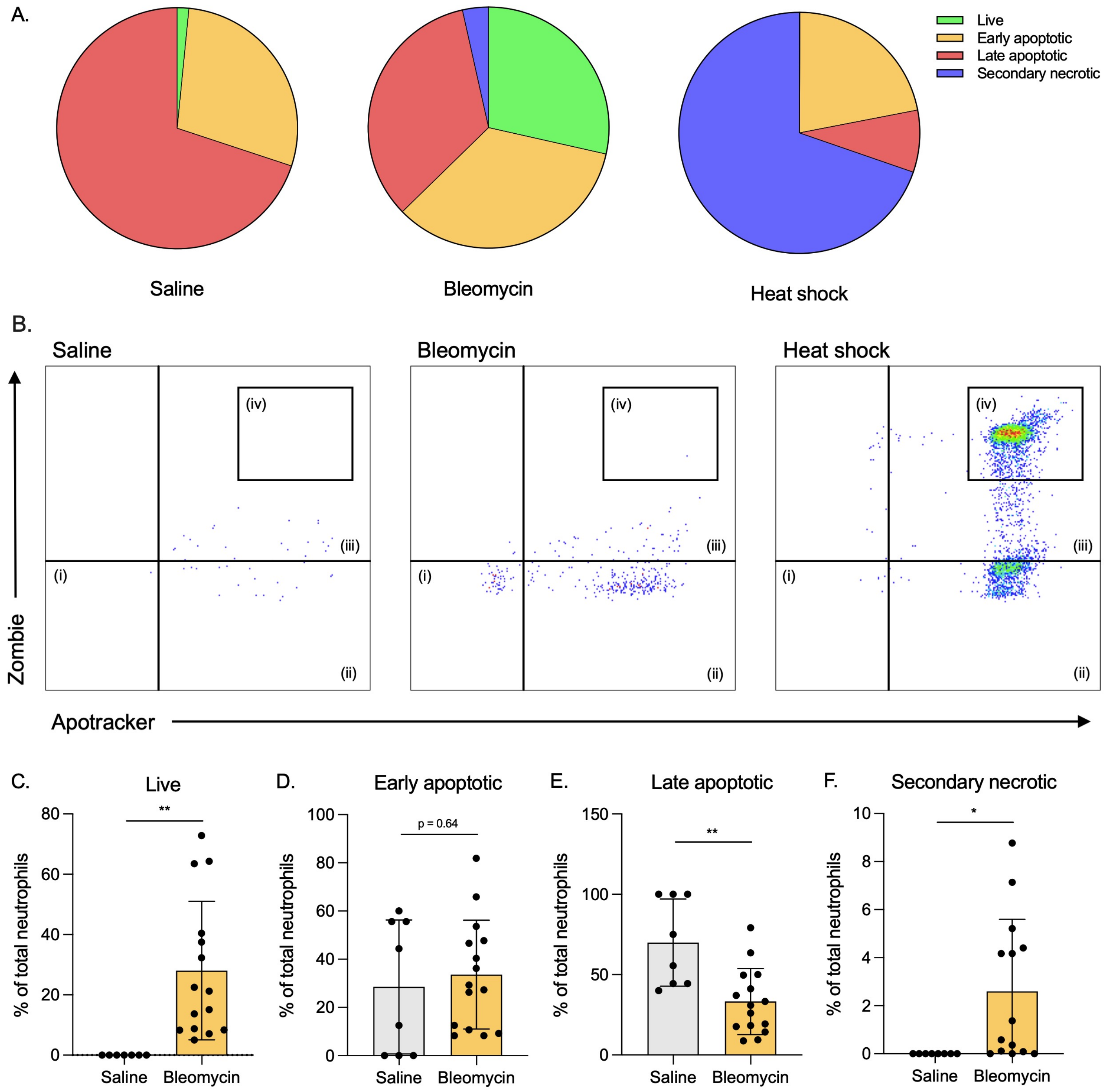

Supplemental Figure 2: Neutrophils in airspaces of fibrotic mice have features of apoptosis resistance and frank necrosis. A. Cells were collected from BALF of saline or bleomycin-treated mice and live, early apoptotic, late apoptotic, and secondary necrotic neutrophils were measured via flow cytometry. Combined cells from saline and bleomycin-treated mice were heat-shocked at 65°C for 5 minutes as a positive control for cell death. **B.** Representative gating scheme of live/dead neutrophil staining. Live cells are Apotracker$^-$ Zombie$^-$ (i), early apoptotic cells are Apotracker$^+$ Zombie$^-$ (ii), late apoptotic cells are Apotracker$^+$ Zombie^{lo} (iii), and secondary necrotic cells are Apotracker$^+$ Zombie^{hi} (iv). **C-F.** Quantification of live (C), early apoptotic (D), late apoptotic (E), and secondary necrotic (F) BALF neutrophils from saline or bleomycin-treated mice. Cells were gated as described in B. N = 6-15 mice per group. Data from two combined experiments. Statistical analysis by unpaired Student's t test. Error bars represent the means +/- SD.

Downloaded from http://journals.aai.org/immunohorizons/article-supplement/237590/pdf/immunohorizon_2200083_supplemental_1/ by guest on 26 September 2023

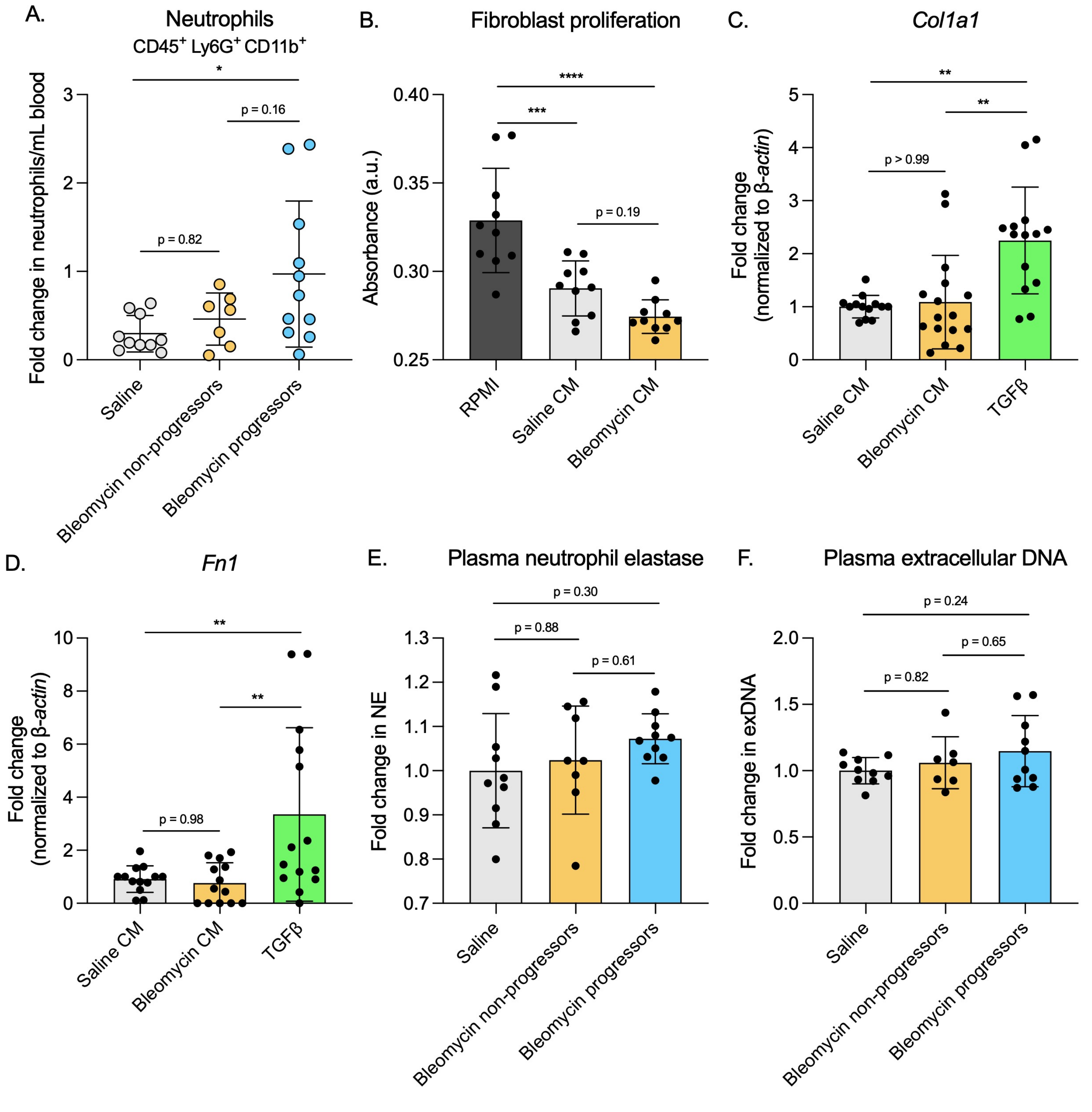

Supplemental Figure 3: No increase in absolute blood neutrophil number or plasma neutrophil products in bleomycin progressor group. A. Cells were collected from blood and stained for neutrophil markers CD45, CD11b, and Ly6G. Neutrophils were quantified using flow cytometry. N = 7-11 mice per group. Data from two combined experiments. Statistical analysis by one-way ANOVA with Tukey's multiple comparisons; error bars represent the means +/- SD. **B.** Fibroblast proliferation as measured by MTT assay. MTT reagent was added to fibroblasts after 24 hours of culture. After 48 hours of culture, proliferation was measured. Representative of 3 independent experiments. N = 1 mouse per group, dots represent technical replicates. Statistical analysis by one-way ANOVA. **C-D.** Fibroblast expression of *Col1a1* (B) and *Fn1* (C) as measured by RT-qPCR following treatment with supernatant from cultured neutrophils isolated from fibrotic (bleomycin CM) or nonfibrotic (saline CM) mice. Neutrophils were cultured *ex vivo* for 4 hours in plain RPMI, after which, supernatant was removed for assays. Fibroblasts were cultured for in complete RPMI or saline and bleomycin CM + BSA + L-glutamine + penicillin/streptomycin + amphotericin. Data from 3 combined experiments. N = 3 mice per group, dots represent technical replicates. Statistical analysis by Kruskal-Wallis test (B) or one-way ANOVA with multiple comparisons (C). **E-F.** NE (E) and exDNA (F) levels in plasma collected from mice described in Figure 6. N = 7-10 mice per group. Data from two combined experiments. Statistical analysis by one-way ANOVA with Tukey's multiple comparisons; error bars represent the means +/- SD.

Downloaded from http://journals.aai.org/immunohorizons/article-supplement/237590/pdf/immunohorizon_2200083_supplemental_1/ by guest on 26 September 2023

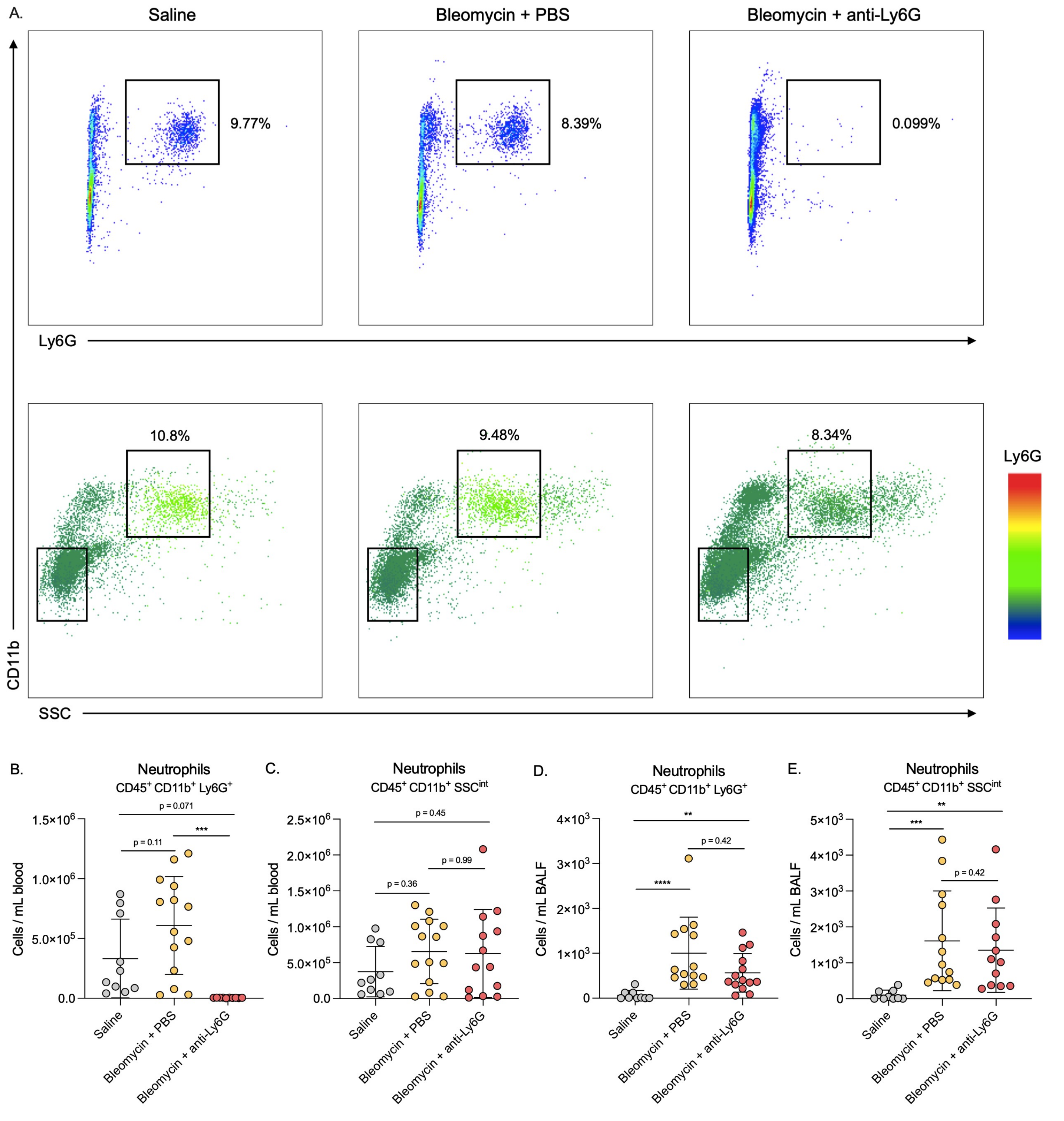

Supplemental Figure 4: Anti-Ly6G treatment does not deplete neutrophils in fibrotic mice. A. Neutrophils from saline, bleomycin + PBS, and bleomycin + anti-Ly6G-treated mice as gated by CD11b and Ly6G or CD11b and SSC. Heat map shows relative expression of Ly6G. **B.** Neutrophils from blood of mice described in A, identified by gating on $CD11b^{+}$ and $Ly6G^{+}$ cells. **C.** Neutrophils from blood of mice described in A, identified by gating on $CD11b^{+}$ SSC^{int} cells. **D.** Neutrophils from BALF quantified as described in B. **E.** Neutrophils in BALF quantified as described in C. For all: N = 10-14 mice per group. Data from two combined experiments; error bars represent the means +/- SD. For B-C: Statistical analysis by one-way ANOVA with Tukey's multiple comparisons. For D-E: Statistical analysis by Kruskal-Wallis test with Dunn's multiple comparisons.

Downloaded from http://journals.aai.org/immunohorizons/article-supplement/237590/pdf/immunohorizon_2200083_supplemental_1/ by guest on 26 September 2023
